# Comparison of urinary adiponectin in the presence of metabolic syndrome in peri- and postmenopausal women

**DOI:** 10.1186/s12905-022-01655-8

**Published:** 2022-03-14

**Authors:** Patsama Vichinsartvichai, Rattana Teeramara, Titima Jirasawas, Prirayapak Sakoonwatanyoo

**Affiliations:** 1 Department of Obstetrics and Gynecology, Faculty of Medicine Vajira Hospital, Navamindradhiraj University, 681 Samsen Rd, Dusit, Bangkok, 10300 Thailand; 2 Department of Clinical Pathology, Faculty of Medicine Vajira Hospital, Navamindradhiraj University, Bangkok, 10300 Thailand

**Keywords:** Urinary adiponectin, Metabolic syndrome, Menopause, Menopausal transition

## Abstract

**Objectives:**

To find the association between urinary adiponectin and metabolic syndrome (MetS) in peri- and postmenopausal women and its potential application as a noninvasive screening for MetS.

**Methods:**

A cross-sectional study was conducted in healthy peri- and postmenopausal women (defined by STRAW + 10 staging) aged at least 40 years who attended annual check-ups or menopause clinics were recruited. Baseline demographic data, MENQOL, anthropometric measurements, blood pressure, laboratory (FBS, total cholesterol, HDL-C, LDL-C, TG), and urinary adiponectin were collected. The MetS was diagnosed according to JIS 2009.

**Results:**

290 peri- and postmenopausal women had participated. The prevalence of Mets among our participants was 18%. Urinary adiponectin levels were similar in peri- and postmenopausal women with and without MetS (2.6 ± 2.2 vs. 2.3 ± 1.9 ng/mL, respectively, *P* = 0.55). Urinary adiponectin provides no diagnostic value for MetS (AUC = 0.516).

**Conclusions:**

Urinary adiponectin has no role in screening and diagnosing MetS in peri- and postmenopausal women. The quest toward noninvasive screening for MetS is still going on.

## Introduction

Women around the world increase their life expectancy [1]. With the finite fertile years, they have spent at least one-third of their life in menopause [2]. The incidence of metabolic syndrome (MetS) and cardiovascular disease (CVD) rise steeply during the menopausal transition [3]. MetS is a combination of multiple metabolic disorders such as visceral adipose tissue accumulation, abnormal fasting plasma glucose, abnormal lipid metabolism, and blood pressure regulation [4]. The menopause-induced estrogen deficiency contributes to body fat pattern redistribution toward visceral fat accumulation [5], insulin resistance, and chronic inflammation [6–8] which contribute to the occurrence of MetS [9].

MetS is considered as the sentinel event before CVD. Many clinical and biochemical markers correlated with MetS and CVD in menopausal women such as premature (age < 40 years) or early (age 40–45 years) at the onset of menopause [10–12], type of menopause (natural, surgical) [13], frequent hot flushes [14–17], early onset of hot flushes (age < 42 years) [15–18], inflammatory biomarkers [CRP, IL-6, and homocysteine-like Lp(a)] [8, 19], and visceral fat biomarkers (adiponectin, leptin, ghrelin) [20].

The visceral fat secretes many hormones involved in MetS such as adiponectin, leptin, and ghrelin [21]. Adiponectin consists of 244-amino acid, and secretes exclusively by adipocytes. Adiponectin is inversely correlated with visceral fat [22]. It exerts its affect through transmembrane receptors AdipoR1, AdipoR2 [23], and a surface membrane receptor T-cadherin (T-Cad) [24]. Adiponectin is a vasoactive peptide that exerts anti-diabetic, anti-atherosclerotic, anti-obesity, and anti-inflammatory effects [25]. It prevents metabolic deterioration toward MetS and expresses cardioprotection. Serum adiponectin is negatively correlated with MetS and could be used as a biomarker for MetS [20]. In our previous study, we reported the diagnostic performance of serum adiponectin for screening of MetS in peri- and postmenopausal women and found that serum adiponectin performs moderately well in the screening of MetS [20]. Adiponectin can be filtered out into the glomerular basement membrane in kidneys and excreted into urine [25, 26].

To the best of our knowledge, there has not been any validation study of urinary adiponectin for screening of MetS. We would like to find the association between urinary adiponectin and MetS in peri- and postmenopausal women and explore its potential application as a noninvasive, in-home screening for MetS.

## Methods

### Study design and participants

Healthy peri- and postmenopausal women (defined by STRAW + 10 stage of reproductive aging [27], which is the international standard to define reproductive aging into 10 stages) aged at least 40 years old (the age that defined premature menopause [28]) who attended an annual health check-up at check-up clinic, or a visit at menopause clinic at a university hospital, were recruited during January–December 2020. We excluded the participants who had a history of stroke, cardiovascular disease, cancer, polycystic ovary syndrome, diagnosed with any inflammatory diseases (SLE, autoimmune disease, rheumatoid arthritis, etc.), on immunosuppressive therapy, steroid or NSAIDs, and chronic kidney disease. The study was conducted following the Declaration of Helsinki, and the study protocol was approved by the Vajira Institutional Review Board. Informed consent was obtained from all participants.

### Outcome measures

All participants have undergone a clinical and biochemical evaluation. The anthropometric measurements (waist circumference, hip circumference, and height) were carried out according to the World Health Organization recommendations [29]. Weight was measured in kilograms. The waist-hip ratio (WHR) was calculated and stratified into android (WHR ≥ 0.85) and gynoid (WHR < 0.85) body fat distribution pattern. The body mass index (BMI) was calculated and stratified into normal (BMI < 23.0 kg/m^2^), overweight (BMI 23.0–29.9 kg/m^2^), and obese (BMI ≥ 30.0 kg/m^2^) [30]. Height was measured while standing in light clothes without footwear. The standard sphygmomanometer was placed at the same level as the participants’ chest for blood pressure measurement.

Afterward, a two-part questionnaire was self-administered. The first part comprised demographic data including age, lifestyle (alcohol consumption, eating habits, and smoking), menstrual history, marital status, parity, education, occupation, and family history of metabolic diseases. The second part was the Thai version of MENQOL questionnaire. The MENQOL was translated and validated at our institution, with Cronbach’s alpha = 0.8940 [31]. The MENQOL questionnaire consists of 29 items within four domains, vasomotor (3 items), psychosocial (7 items), physical (16 items), and sexual (3 items). The participants were demanded to rate their experience of each of the items within the previous month and to score the bothersome of each symptom on a Likert scale ranging from 0 (not disturbed at all) to 6 (extremely disturbed). The investigators supervised the self-administered questionnaire or interviewed and completed the questionnaire for illiterate participants.

After an overnight fast, the blood specimen was drawn for bio-chemical evaluations including complete blood count (CBC), fasting blood glucose (FBG), triglyceride (TG), total cholesterol, high-density lipoprotein cholesterol (HDL-C), and low-density lipoprotein cholesterol (LDL-C). The biochemical assays were conducted in an ISO 15189 certified biochemical laboratory at the department of clinical pathology. The FBG, total cholesterol, HDL-C, and TG were analyzed with an auto-analyzer (SIEMENS Dimension® EXL™ 200, USA) and reported as mg/dL. LDL-C was calculated using the Friedewald equation and reported as mg/dL.

Urine adiponectin was measured by an ultrasensitive human adiponectin ELISA kit (Invitrogen, Thermo Fisher Scientific, Austria) [32] with an auto-analyzer (TECAN® SUNRISE, Austria). Urine samples were collected and transferred to pyrogen/endotoxin-free tubes, and then snap-frozen at – 20 °C for further analysis according to the manufacturer’s recommendation. Each sample was assayed in duplicate with tenfold dilution using the quantitative sandwich enzyme immunoassay technique. The range of measured concentrations is 0–32 ng/mL using diluted reconstituted standard human adiponectin according to the manufacturer protocol. The coefficient variation (%CV) of intra- and inter-assay were less than 8.31% and 9.69%, respectively.

### Criteria for diagnosis of MetS

The diagnosis of MetS was made following the Joint Interim Statement 2009 (JIS 2009) criteria [33], where the participants presented at least three of the following: (1) abdominal obesity defined as waist circumference ≥ 80 cm for Asian women; (2) elevated TG ≥ 150 mg/dl or drug treatment for elevated triglycerides; (3) reduced HDL-C < 50 mg/dl or drug treatment for reduced HDL-C; (4) elevated blood pressure defined as systolic ≥ 130 mmHg and/or diastolic ≥ 85 mmHg or antihypertensive drug treatment; and (5) elevated fasting glucose ≥ 100 mg/dl or drug treatment of elevated glucose.

### Statistical analysis

We applied the formulae for sample size estimation in diagnostic test studies of biomedical informatics for adequate sensitivity/specificity [34] i.e. urinary adiponectin for diagnosis of MetS. From our previous study [8], we found that the prevalence of metabolic syndrome in peri- and postmenopausal women was 21.4%. With α = 0.05, and 80% power, two hundred and ninety participants were required for this study.

All data were analyzed using IBM SPSS statistics version 22.0 (SPSS Inc., USA). Data were presented as mean ± SD, number (%), or percentage (95% confidence interval—CI), as appropriate. Urinary adiponectin was analyzed and compared among participants with and without MetS using independent sample *t*-test or one-way ANOVA as appropriate. Pearson’s correlation coefficient was determined for the correlation between urinary adiponectin and MetS components. The area under the curve (AUC) of receiver operating characteristic (ROC) curve analysis for diagnosing MetS was performed to obtain the diagnostic performance and cutoff of urinary adiponectin for diagnosis of MetS by Yuden index. The *P* value of < 0.05 was considered statistically significant.

## Results

290 peri- and postmenopausal women aged 57.2 ± 8.2 years were recruited. The prevalence of MetS among our participants was 18% (55 in 290 participants). Baseline characteristics and demographic information of participants were presented in Table 1. The participants with MetS were slightly older age and more into their menopausal years; moreover, they had worse health habits (alcohol drinking, fatty food, less exercise).

**Table 1:** Baseline characteristics of the participants (N = 290)

Characteristics	MetS (n = 55)	No MetS (n = 235)
Age (years)	57.2 ± 8.2	54.3 ± 8.3
*Menopausal status, n* (%)		
Perimenopause	17 (30.9)	83 (35.3)
Postmenopause	38 (69.21)	152 (64.7)
*Level of highest education, n* (%)		
Elementary school	16 (29.1)	37 (15.7)
High school	14 (25.5)	44 (18.7)
Undergrad or higher	25 (45.5)	151 (64.3)
*Lifestyle behaviors, n* (%)		
Current alcohol drinker	5 (9.1)	7 (3.0)
Preferred of fatty food	30 (54.7)	76 (32.3)
Regular exercise	14 (25.5)	104 (44.3)
Current smoker	0 (0)	6 (2.6)

*Independent sample t-test

**Chi-square

**Fig. 1:**
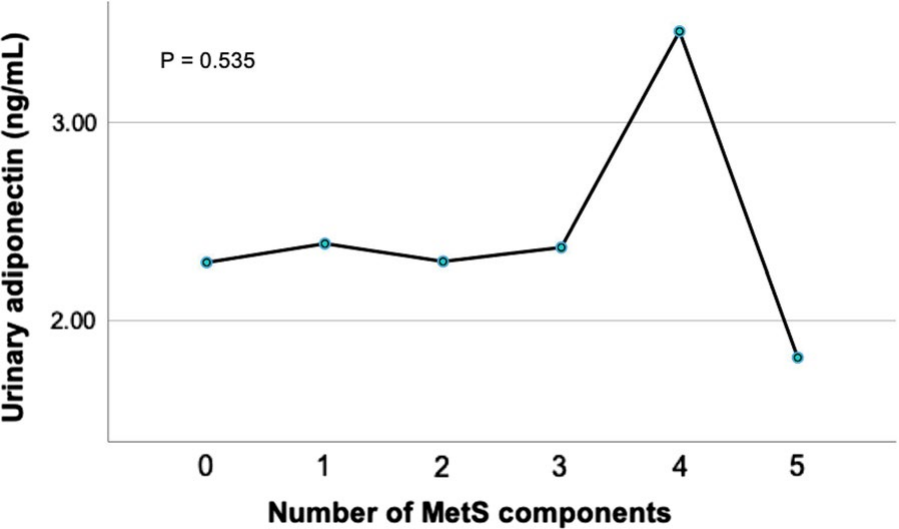
The mean urinary adiponectin (ng/mL) among participants without any components of MetS and those with 1–5 components of diagnostic criteria were statistically similar (*P* = 0.535)

**Fig. 2:**
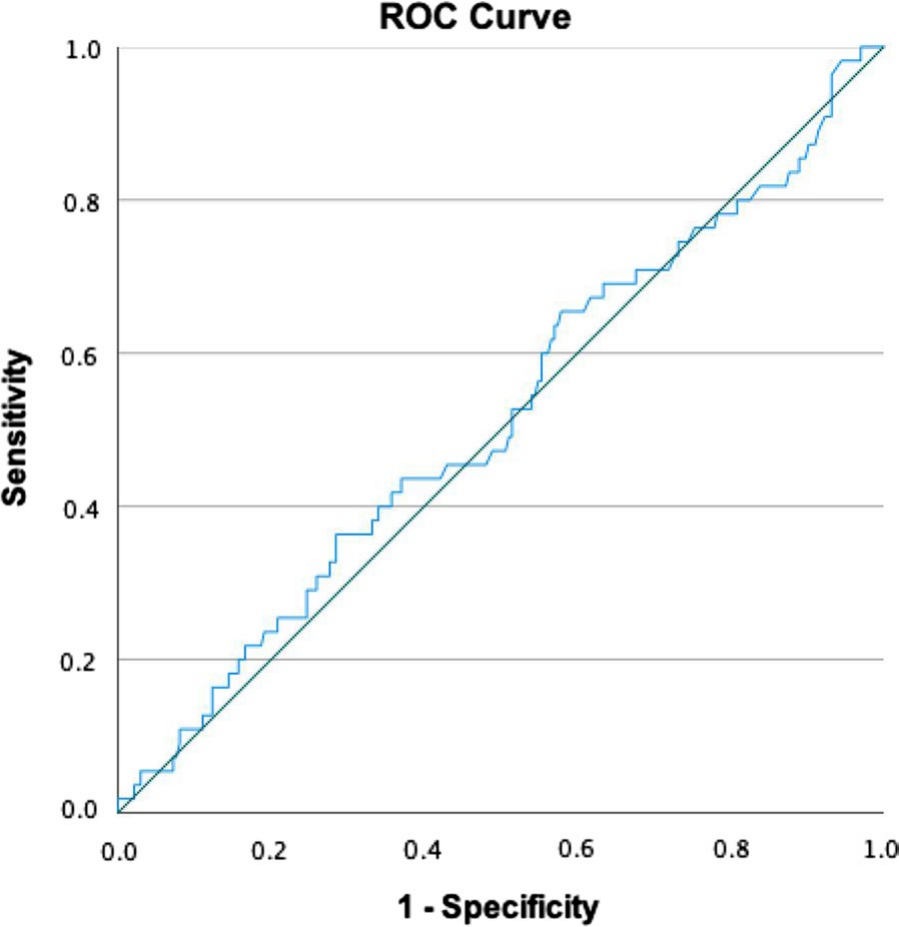
ROC curve of urinary adiponectin in the diagnosis of MetS. The area under the curve is 0.516 which represents no diagnostic value of urinary adiponectin for MetS

Urinary adiponectin level in participants with MetS group was slightly higher than participants without Mets but did not reach statistical significance (2.6 ± 2.2 vs 2.3 ± 1.9 ng/mL, respectively, *P* = 0.55). Urinary adiponectin also stabled with the increment of component of MetS in our participants (Fig. 1). Urinary adiponectin had negative correlations with waist circumference, body weight, BMI, FBG, TG, total cholesterol LDL-C, and HDL-C. The comparison of anthropometric measurements and metabolic profiles was presented in Table 2. Urinary adiponectin could not discriminate peri- and postmenopausal with and without MetS. The ROC curve of urinary adiponectin was presented in Fig. 2.

**Table 2:** Anthropometric measurements and metabolic profile of the participants (N = 290)

Characteristics	MetS (n = 55)	No MetS (n = 235)	*P* value*
Urine adiponectin (ng/mL)	2.6 ± 2.2	2.3 ± 1.9	0.55
Systolic BP (mmHg)	134.2 ± 9.9	122.5 ± 13.3	< 0.001
Diastolic BP (mmHg)	80.6 ± 13.7	75.1 ± 10.9	0.02
*Anthropometric measurements*			
Body weight (kg)	64.4 ± 9.3	56.3 ± 9.6	< 0.001
Height (cm)	155.3 ± 5.3	155.6 ± 6.9	0.79
BMI (kg/m^2^)	26.8 ± 3.9	23.1 ± 3.5	< 0.001
Waist circumference (cm)	87.6 ± 7.8	78.2 ± 9.2	< 0.001
Hip circumference (cm)	102.5 ± 9.2	95.9 ± 7.9	< 0.001
Waist-to-hip ratio (WHR)	0.85 ± 0.1	0.81 ± 0.1	< 0.001
Overweight and obesity, n (%)	36 (65.5)	53 (22.5)	< 0.001
Abdominal obesity, n (%)	50 (90.5)	86 (36.6)	< 0.001
*Body fat distribution pattern, n* (%)			
Gynoid (WHR < 0.85)	29 (52.7)	187 (79.6)	< 0.001
Android (WHR ≥ 0.85)	26 (47.3)	48 (20.2)	
*Metabolic profiles*			
Fasting blood sugar (mg/dL)	114.1 ± 34.4	93.9 ± 8.6	< 0.001
Total cholesterol (mg/dL)	237.2 ± 44.3	226.6 ± 41.6	0.096
HDL (mg/dL)	54.6 ± 12.9	69.2 ± 15.3	< 0.001
LDL (mg/dL)	154.1 ± 39.6	140.5 ± 35.9	0.014
Triglyceride (mg/dL)	166.6 ± 75.8	94.7 ± 38.0	< 0.001

*Independent sample t-test

**Chi-square

**Fig. 3:**
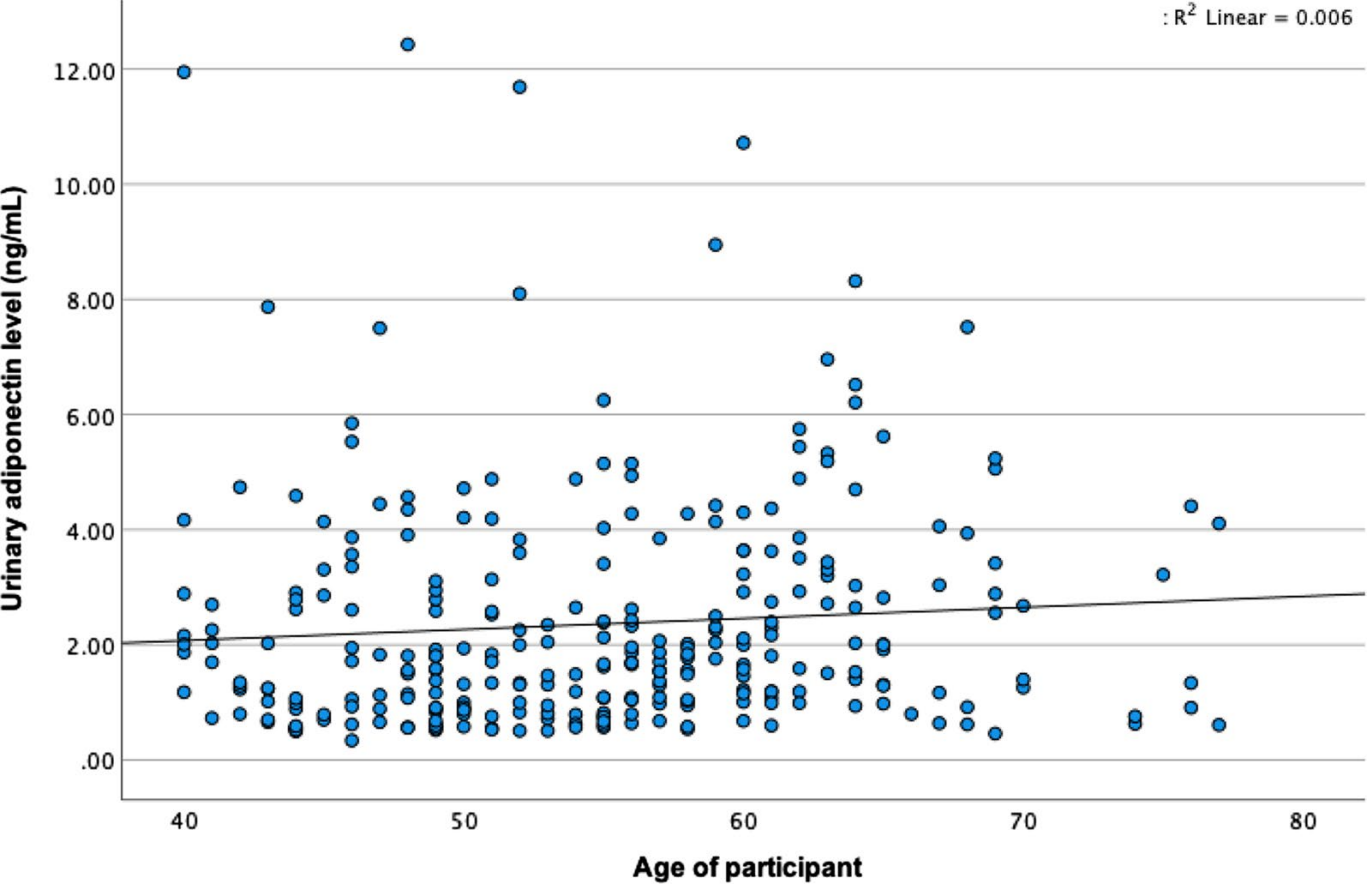
Scattered plot showing urinary adiponectin level slowly increased with the age of participants but not significantly correlated (R^2^ = 0.006, *P* = 0.174)

Urinary adiponectin increased slowly with age after the late postmenopause period (Table 3) but did not change during the menopausal transition (Fig. 3). However, the urinary adiponectin did not significantly correlate with age, R^2^ = 0.006, *P* = 0.174 (Fig. 3).

**Table 3:** Urinary adiponectin stratified by age group (N = 290)

	40–49 years (n = 90)	50–59 years (n = 111)	60–69 years (n = 78)	70–79 years (n = 11)	*P* value*
Urinary adiponectin (ng/ml)	2.2 ± 2.2	2.2 ± 1.8	2.9 ± 2.0	2.0 ± 1.4	0.041

*One-way ANOVA

The quality of life in peri- and postmenopausal women with and without Mets were similar across each domain (Table 4). However, the global quality of life was better in participants without MetS than those with MetS.

**Table 4:** MENQOL among participants (N = 290)

MENQOL	MetS (n = 55)	No MetS (n = 235)	*P* value*
Vasomotor domain	2.6 ± 1.5	2.1 ± 1.3	0.022
Psychological domain	2.7 ± 1.2	2.3 ± 1.2	0.059
Physical domain	3.2 ± 1.1	2.9 ± 1.1	0.045
Sexual domain	4.3 ± 2.9	3.4 ± 2.8	0.042
Global quality of life	3.1 ± 1.0	2.7 ± 1.0	0.007

*Independent sample t-test

## Discussion

Although the urinary adiponectin increased with age, our study failed to find its association with MetS in peri- and postmenopausal women. To the best of our knowledge, this is the first study to explore the potential use of ultrasensitive urinary adiponectin for MetS.

Previous studies explored the use of urinary adiponectin for the screening or early detection of microvascular injury in diabetes mellitus [26, 35], and SLE [36], glomerular injury, and proteinuria in IgA nephropathy [37]. Furthermore, another study found that high urinary adiponectin levels were associated with the severity of arterial stiffness and have a positive correlation with FBG, TG, and blood pressure [38]. In those studies, urinary adiponectin performed quite well to detect the injury of microvasculature or glomerular unit. The advanced progression of the disease and a higher degree of microvascular injury increase the excretion of urinary adiponectin [26, 35–37]. The microvascular injury in our cohort (peri- and postmenopausal women with MetS) might be minimal because of the early detection and treatment of MetS before the occurrence of metabolic diseases, and CVD might delay the microvascular injury [39]. Monitoring the changes of urinary adiponectin over the menopausal transition or during the development of MetS until cardiovascular event might help us confirm our hypothesis in the future.

In our participants, urinary adiponectin was not different in the early stage of metabolic disease compared to healthy participants. However, the urinary adiponectin in women with and without MetS might be different in other races where lifestyle, body habitus, and gene expression profile, which need to evaluate in further investigation.

The quality of life in peri- and postmenopausal women with and without MetS in this study were similar in every domain unlike our previous survey [5], where those women with MetS or android body fat distribution pattern performed poorer in the vasomotor and psychological domain. This finding is explained by the age of participants. In this study, women with MetS were significantly older than women without MetS this study while the average age between the two groups was similar in our previous study. The severity of vasomotor symptoms can relieve spontaneously over time without any treatment [40]. In other words, our participants with MetS might pass the early postmenopausal years for a while so they had fewer vasomotor symptoms.

## Conclusions

Urinary adiponectin level is not different in the presence of MetS in peri- and postmenopausal women. Further investigation should focus on the other marker that can potentially use as a noninvasive screening test for MetS. The study of urinary adiponectin in peri- and postmenopausal women is still warrant further investigation to see its changes during menopausal transition or the progression of metabolic diseases toward cardiovascular events.

## Data Availability

The datasets generated during and/or analyzed during the current study are not publicly available due to informed consent and confidentiality but are available from the corresponding author on reasonable request.
